# Trends in non-metastatic prostate cancer management in the Northern and Yorkshire region of England, 2000–2006

**DOI:** 10.1038/sj.bjc.6605424

**Published:** 2009-11-10

**Authors:** L Fairley, M Baker, J Whiteway, W Cross, D Forman

**Affiliations:** 1 Northern and Yorkshire Cancer Registry and Information Service, Level 6, Bexley Wing, St James's Institute of Oncology, St James's Hospital, Beckett Street, Leeds, LS9 7TF, UK; 2 Leeds Teaching Hospitals Trust, Bexley Wing, St James's Institute of Oncology, St James's Hospital, Leeds, LS9 7TF, UK; 3 North of England Cancer Network, Team View, Fifth Avenue Business Park, Team Valley Trading Estate, Gateshead, Tyne and Wear, NE11 OXA, UK; 4 Pyrah Department of Urology, St James's Hospital, Beckett Street, Leeds, LS9 7TF, UK; 5 Cancer Epidemiology Group, Centre for Epidemiology and Biostatistics, University of Leeds, Level 6, Bexley Wing, St James's Institute of Oncology, St James's Hospital, Beckett Street, Leeds, LS9 7TF, UK

**Keywords:** prostate cancer, radical prostatectomy, brachytherapy, radiotherapy, treatment variation

## Abstract

**Background:**

Our objective was to analyse variation in non-metastatic prostate cancer management in the Northern and Yorkshire region of England.

**Methods:**

We included 21 334 men aged ⩾55, diagnosed between 2000 and 2006. Principal treatment received was categorised into radical prostatectomy (11%), brachytherapy (2%), external beam radiotherapy (16%), hormone therapy (42%) and no treatment (29%).

**Results:**

The odds ratio (OR) for receiving a radical prostatectomy was 1.53 in 2006 compared with 2000 (95% CI 1.26–1.86), whereas the OR for receiving hormone therapy was 0.57 (0.51–0.64). Age was strongly associated with treatment received; radical treatments were significantly less likely in men aged ⩾75 compared with men aged 55–64 years, whereas the odds of receiving hormone therapy or no treatment were significantly higher in the older age group. The OR for receiving radical prostatectomy, brachytherapy or external beam radiotherapy were all significantly lower in the most deprived areas when compared with the most affluent (0.64 (0.55–0.75), 0.32 (0.22–0.47) and 0.83 (0.74–0.94), respectively) whereas the OR for receiving hormone therapy was 1.56 (1.42–1.71).

**Conclusions:**

This study highlights the variation and inequalities that exist in the management of non-metastatic prostate cancer in the Northern and Yorkshire region of England.

Prostate cancer is the most commonly diagnosed cancer in men in the United Kingdom, accounting for nearly a quarter of all new male cancer diagnoses (Cancer Research UK, 2009). Prostate cancer incidence has increased dramatically over the post 20 years (Majeed *et al*, 2000; Cancer Research UK, 2009) and much of this is because of prostate-specific antigen (PSA) testing, which has resulted in many diagnoses of prostate cancer that would not have previously been diagnosed (Brewster *et al*, 2000; Evans and Moller, 2003). Prostate cancer is also the most commonly diagnosed cancer in men throughout Europe (Ferlay *et al*, 2007) and the United States (SEER, 2009).

Treatments for prostate cancer include radical prostatectomy, radiotherapy (including both brachytherapy and external beam radiotherapy), hormone therapy, active surveillance and watchful waiting. Radical treatments, such as surgery and radiotherapy, can have serious side effects that affect quality of life, such as urinary, bowel and sexual dysfunction. Side effects for hormone treatment include osteoporosis, cardiovascular disease, loss of libido and gynaecomastia. There is no consensus on the best treatment for early prostate cancer (Selley *et al*, 1997; NICE, 2002, 2008). Improving Outcomes Guidance for Urological Cancers published in 2002 by the National Institute for Clinical Excellence (NICE) (NICE, 2002) recognised that although observation studies suggest that radical treatments can improve long-term survival, these studies are subject to bias in the selection of particular patient groups. Information on treatment complications and quality of life after radical treatment must also be taken into consideration when assessing treatment options. NICE published clinical guidelines for the diagnosis and treatment of prostate cancer in 2008 (NICE, 2008). Selection of treatment for men with localised prostate cancer is based on their risk that is derived from a combination of PSA levels, Gleason score and clinical stage. For example, for men with low-risk localised prostate cancer, the preferred treatment option, as recommended by NICE, is active surveillance; however, other treatment options include prostatectomy, brachytherapy or conformal radiotherapy. However, in clinical practice, treatment decisions are more complex and various studies in the United Kingdom have found that there were wide variations in the management of prostate cancer (Savage *et al*, 1997; Donovan *et al*, 1999; Hanna *et al*, 2002; Payne and Gillatt, 2007).

Socio-demographic factors that are found to be associated with the treatment received for prostate cancer include ethnicity, socio-economic status, income and geographical area of residence (Harlan *et al*, 2001; Bauvin *et al*, 2003; Cooperberg *et al*, 2004; Krupski *et al*, 2005). Results show that patients with low-income or low socio-economic status are less likely to receive surgery or radiotherapy.

In this paper we describe the variation and management of non-metastatic prostate cancer using data from a population-based cancer registry. We explore the trends between 2000 and 2006 and analyse what factors are associated with treatment received.

## Materials and methods

The Northern and Yorkshire Cancer Registry and Information Service (NYCRIS) is a population-based cancer registry covering a population of approximately 6.7 million people, collecting data on all new diagnoses of cancer within its population. Information collected includes tumour, demographic and treatment information. Data for this analysis were extracted from the NYCRIS database. There were 24 946 men diagnosed with prostate cancer between 2000 and 2006, aged ⩾55 at diagnosis. Men with metastases, including distant nodal involvement, at diagnosis were excluded from the analysis (*n*=3281). Men who were registered from a death certificate only and men who had a survival time of zero days (*n*=224) were also excluded from analysis.

The Northern and Yorkshire Cancer Registry and Information Service NYCRIS records treatment planned at diagnosis, and therefore, men on active surveillance or watchful waiting will have no treatment recorded in the cancer registry database although they may go on to receive treatment in the future. From our data we identified the principal treatment received using five different treatment categories: radical prostatectomy, brachytherapy, external beam radiotherapy, hormone treatment and no treatment. A further 103 men were excluded from the study as they did not fit into any of the treatment categories listed; some had chemotherapy recorded as the treatment received and others had different surgery codes recorded. We have included a no treatment group as this consisted of a large percentage of the study population (29%), although this group includes a mix of patients on active surveillance, watchful waiting and those who actually receive no treatment and we cannot distinguish between these three groups from the data that we have. This resulted in a study population of 21 334 men.

Other explanatory variables extracted for each patient were age at diagnosis, year of diagnosis and postcode of residence at diagnosis. This postcode was then used to assign patients to a cancer network of residence and deprivation score. An area-based measure of deprivation, the Index of Multiple Deprivation 2004 (Noble *et al*, 2004), was used to measure socio-economic status. The Income domain of this index was used in which each patient was assigned a score based on their postcode and these scores were ranked and split into quintiles: quintile 1 contains patients in the most affluent areas and quintile 5 contains patients in the most deprived areas. Patients were also assigned to a Cancer Network based on their postcode of residence at diagnosis. Cancer networks bring together health service commissioners and providers, the voluntary sector and local authorities and are responsible for the delivery of cancer services within a geographical area. There are currently three cancer networks covering the NYCRIS region and these have been used in the analysis presented here: Yorkshire Cancer Network (YCN), Humber and Yorkshire Coast Cancer Network (HYC) and North of England Cancer Network (NECN).

Age-standardised rates per 100 000 population for all ages were calculated using the European Standard Population (Office for National Statistics, 1998). Univariate tests of association between the explanatory variables and the principal treatment received were carried out using *χ*^2^ test. Multivariate logistic regression was then used to examine the associations between the explanatory variables and each mode of treatment received.

## Results

Overall, the age-standardised incidence rate, standardised to the European standard population for all ages, was 93.5 per 100 000 population, and this increased from 83.1 per 100 000 in 2000 to 96.6 per 100 000 in 2004 (Table 1). There were a total of 21 334 cases in our study population after exclusions and the number of these cases diagnosed each year increased from 2572 in 2000 to 3264 in 2006.

**Figure 1:**
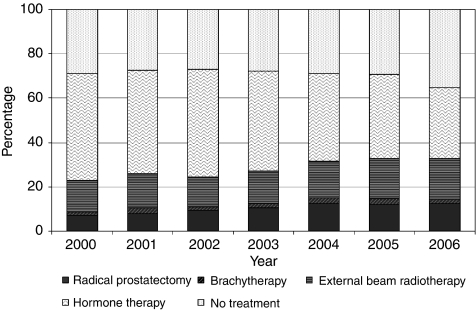
Principal treatment by year of diagnosis.

Table 2 shows the overall percentage of men receiving each treatment. Table 3 shows the demographic characteristics of the study population by treatment group and Figure 1 shows the principal treatment by year of diagnosis.

Overall, between 2000 and 2006, hormone therapy was the most common mode of treatment (42%), whereas 29% of patients received no treatment and 11% of patients received a radical prostatectomy. The rate of radical prostatectomy increased from 7% in 2000 to 13% in 2006. The brachytherapy rate remained at approximately 2% over the 7-year period and the external beam radiotherapy rate increased slightly over time from 14% in 2000 to 18% in 2006. The use of hormone therapy decreased over time from 48% in 2000 to 32% in 2006. The percentage of patients receiving no treatment remained between 27 and 29% from 2000 to 2005 but increased to 35% in 2006.

There were higher percentages of men aged 55–64 and 65–74 years who received all three radical treatments when compared with men aged ⩾75 years; for men aged ⩾75 years, 3% received a radical prostatectomy, 1% received brachytherapy and 8% received hormone therapy. Lower percentages of men aged 55–64 years received hormone therapy (8%) and no treatment (14%) when compared with men aged ⩾65 years.

There were statistically significant differences in the treatments received by network of residence. Radical prostatectomies were more common in YCN (14%) than in HYC (8%) and in NECN (9%). Brachytherapy was also more common in YCN (3%) than the other two networks. External beam radiotherapy was more common in YCN (18%) and in HYC (22%) than in NECN (12%). Only 35% of patients in YCN were treated by hormone therapy compared with 43% of patients in HYC and 48% in NECN. Fewer men in HYC (25%) received no treatment when compared with YCN (29%) and NECN (31%).

**Figure 2:**
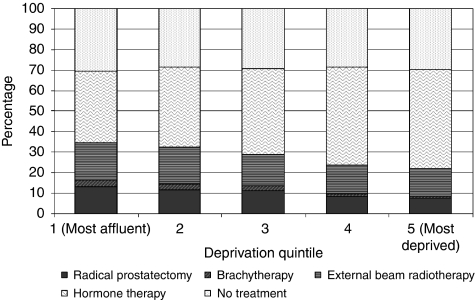
Principal treatment by deprivation quintile.

The treatment received also varied by deprivation quintile. There was a linear trend in the percentage of men who received a radical prostatectomy: 13% of men in the most affluent areas received this treatment when compared with only 8% of men in the most deprived areas. A similar socio-economic gradient was also observed for brachytherapy and external beam radiotherapy: rates were highest in the most affluent areas and lowest in the most deprived areas. Only 35% of men in the most affluent areas received hormone therapy compared with 48% of men in the most deprived areas. There was not much difference in the percentage of men who received no treatment by deprivation quintile. Figure 2 also shows the principal treatment received by deprivation quintile.

Table 4 shows the results from the multivariate logistic regression models.

Men diagnosed with prostate cancer in 2006 were 53% more likely to receive a radical prostatectomy than men diagnosed in 2000 (odds ratio (OR)=1.53, 95% confidence interval (CI) 1.26–1.86). There were no statistically significant differences in the likelihood of receiving brachytherapy or external beam radiotherapy over time. Men diagnosed in 2006 were 43% less likely to have hormone treatment than men diagnosed in 2000 (OR=0.57, 95% CI 0.51–0.64). The odds of having no treatment were 42% higher in 2006 when compared with 2000 (OR=1.42, 95% CI 1.27–1.59).

There was a very strong association between age and treatment received. The odds of having a radical prostatectomy, brachytherapy or external beam radiotherapy decreased as age at diagnosis increased. The OR for men aged ⩾75 years, compared with men aged 55–64 years, for receiving a radical prostatectomy were 0.02 (95% CI 0.01–0.02), for receiving a brachytherapy were 0.01 (95% CI 0.004–0.03) and for receiving an external beam radiotherapy were 0.08 (95% CI 0.07–0.10). The odds of receiving hormone therapy, and no treatment significantly increased as age at diagnosis increased. The OR for men aged ⩾75 years, compared with men aged 55–64 years, for receiving hormone therapy were 7.67 (95% CI 7.00–8.41) and for receiving no treatment were 2.15 (95% CI 1.98–2.35).

For all treatment modalities there were statistically significant differences across the networks. Compared with YCN, radical prostatectomy and brachytherapy were less likely in HYC (OR=0.54, 95% CI 0.47–0.63 and OR=0.54, 95% CI 0.40–0.71) and in NECN (OR=0.64, 95% CI 0.58–0.71 and OR=0.36, 95% CI 0.28–0.45). External beam radiotherapy was more likely in HYC (OR=1.35, 95% CI 1.22–1.50) and less likely in NECN (OR=0.58, 95% CI 0.53–0.63) compared with YCN. The odds of receiving hormone therapy were more likely in both HYC and NECN compared with YCN (OR=1.36, 95% CI 1.25–1.49 and OR=1.64, 95% CI 1.53–1.75). The odd of receiving no treatment were significantly lower in HYC than in YCN (OR=0.81, 95% CI 0.74–0.88) and significantly higher in NECN than in YCN (OR=1.09, 95% CI 1.02–1.17).

The odds of receiving a radical prostatectomy, brachytherapy or external beam radiotherapy were all significantly lower in the most deprived areas compared with the most affluent (OR=0.64, 95% CI 0.55–0.75; OR=0.32, 95% CI 0.22–0.47; and OR=0.83, 95% CI 0.74–0.94, respectively). The odds of receiving hormone therapy increased as deprivation increased (OR=1.56, 95%CI 1.42–1.71) for most deprived areas compared with most affluent areas. The association between deprivation and receiving no treatment was of borderline statistical significance (*P*=0.05); the magnitude of the effect was similar in all deprivation quintiles relative to the most affluent quintile but only statistically significantly different in quintiles 2 and 4 (OR=0.90, 95% CI 0.82–0.99 and OR=0.86, 95% CI 0.78–0.95 respectively).

## Discussion

Our results show that there are variations in the modality of treatment received in men with non-metastatic prostate cancer in the Northern and Yorkshire region. Younger men were more likely to receive radical treatments, radical prostatectomy, brachytherapy and external beam radiotherapy, as were men from the more affluent areas, whereas older men and men from the more deprived areas were more likely to receive hormone therapy. There were also differences by cancer network of residence. Over the study period, the rates of radical prostatectomies increased whereas the rate of hormone therapy decreased.

Socio-demographic factors, such as age, ethnicity, geographic region of residence and income, have all been found to be associated with prostate cancer treatment elsewhere, including studies from the United States and France (Harlan *et al*, 2001; Bauvin *et al*, 2003; Cooperberg *et al*, 2004; Krupski *et al*, 2005). We also found significant associations between treatment and age, deprivation and geographic region of residence. Radical treatments were less likely in the older population and men from deprived areas and may be explained by differences in life expectancy, with both these groups having shorter life expectancy (Bajekal, 2005).

### Strengths and limitations of the study

One of the main strength of this study is that it is population based and includes data from over 21 000 cases, and therefore produced statistically robust estimates. It is also recognised that the coding of treatment procedures were of high quality and well recorded with the Northern and Yorkshire Cancer Registry and Information Service (United Kingdom Association of Cancer Registries, 2009).

One of the main limitations of this study is that we do not have data available on stage and grade of the tumours, or PSA levels, as these data items are not routinely collected by NYCRIS. The recently established National Cancer Intelligence Network aims to develop a national cancer data repository, involving linking the cancer registration data with other data sources. Work to link urological data from cancer registries to Hospital Episode Statistics and data from the clinical database of the British Association of Urological Surgeons (BAUS) is underway (Ayres *et al*, 2008) and data sets such as these will provide valuable information on stage and grade so that full case-mix adjustment can be made.

The recent increase in PSA testing, which has been found to be more common in affluent areas (Melia *et al*, 2004; Rowan *et al*, 2008), has resulted in many more cancers being diagnosed earlier and these cancers may be more likely to receive radical treatments. Schröder *et al* (2009) found that PSA-based screening reduced the rate of death from prostate cancer by 20% but was associated with a high risk of diagnosing many men who would not have clinical symptoms during their lifetime.

The other main limitation of this study is regarding the use and interpretation of the no treatment group. This group contains all patients who had no record of receiving any treatment. The routine collection of data within the cancer registry includes treatment intent at diagnosis. The no treatment group in our study will include men on active surveillance and watchful waiting and some of this group may go on to receive some form of treatment; however, this will not be recorded on the cancer registry database. This group will also include patients who actually received no treatment and there may also be some other cases in which we do not know whether the patient received any treatment. Therefore, results concerning this group should be interpreted with caution. We have excluded all records only notified from Death Certificates or with zero survival (0.9% of study population), thus reducing bias from this source.

The percentage of men who received no treatment in 2006 increased to 35% compared with 27–29% between 2000 and 2005. This increase was observed across all cancer networks as more men may be opting for deferred treatment. This increase may just be because of chance and further monitoring of trends is required and when national data become available, trends across other areas can also be analysed.

There are important differences between active surveillance and watchful waiting, although patients on both treatment options are spared the side effects of unnecessary treatment. Active surveillance is an option for men who are suitable for radical treatment but for whatever reasons choose to delay treatment; however, if there are signs of tumour progression, then radical treatment may be offered. Watchful waiting is an option for men not suitable for radical treatments, such as older men or those with poor general health, or some men presenting with asymptomatic advanced disease; if the cancer progresses then these men will generally receive hormone therapy. Our study does not allow us to distinguish between these two groups. However, there was some evidence that men from more deprived areas were less likely to receive no treatment but with borderline significance. Treatment decisions, including the option of active surveillance or watchful waiting, should be made taking into consideration the needs and preferences of the patients and after careful consideration of the options and discussions with their healthcare professionals (NICE, 2008). These decisions could be related to educational levels and the deliberation to choose to delay treatment may be easier and more likely in men from better educated groups. Kane *et al* (2003) found that the education levels of the patients were predictive of primary treatment for prostate cancer but the effect of education depends on age.

### Treatment options

The use of radical prostatectomy increased nearly 20-fold between 1991 and 1999 in England (Oliver *et al*, 2003), and in our study population the odds of having a radical prostatectomy increased significantly by 53% from 2000 to 2006. The rise in surgery may be because of an increase in the numbers of surgeons with the technical ability and enthusiasm to perform this procedure. Surgery is an option for men whose tumours are confined to the prostate and who are expected to live for at least 10 years (NICE, 2002). It can cause significant complications that affect quality of life, such as impotence and incontinence; generally, it will be the fittest men with localised prostate cancer who are selected for surgery. We found a very strong association between age and surgery. We also found that men from the most deprived areas were 36% less likely to have a radical prostatectomy than men from the most affluent areas. Co-morbidity could explain part of this relationship as we would expect greater levels of co-morbidities in men in the more deprived areas and therefore these men will be less likely to be suitable for surgery. However, a study in the Netherlands found that for prostate cancer patients there was no association between socio-economic status and levels of co-morbidity (Schrijvers *et al*, 1997). A study in Western Australia found that men who received radical prostatectomy had less co-morbidities and were more socio-economically advantaged (Hall *et al*, 2005). Although radiotherapy is non-invasive, it shares most of the complications and side effects that follow surgery. Therefore, the extent that co-morbidities may have in explaining the inequalities that we observed in external beam radiotherapy, in which men from more deprived areas were less likely to receive external beam radiotherapy, is not clear.

Brachytherapy is an option suitable for men with smaller prostates who may choose any other radical treatment, including patients who are not suitable for surgery (Ash *et al*, 1998). The use of brachytherapy in general has become more common throughout Europe (Guedea *et al*, 2007). This was the least common mode of treatment received in our study population. We did, however, find a large deprivation gap in the odds of getting brachytherapy; men from the most deprived areas were 68% less likely to receive this form of treatment than men from the most affluent areas after adjustment for age and network, suggesting a lack of access to this treatment for men from socially deprived areas. The odds of receiving brachytherapy were also reduced in older men when compared with younger men. Brachytherapy can be technically challenging in larger prostate glands, although some centres may downsize the gland with hormones before brachytherapy. The prostate gland increases in size with age and hence this form of treatment may not be suitable for older men.

Hormone therapy is the recommended treatment for men with locally advanced prostate cancer either with or without external beam radiotherapy (NICE, 2002). A recent study by Widmark *et al* (2009) found that the addition of local radiotherapy to endocrine treatment halved the 10-year prostate cancer-specific mortality. Hormone therapy was the most common form of treatment received in our study population and was more common among older men and men from more deprived areas. This may suggest that men from deprived areas present with more advanced cancers and that radical treatment might not be suitable for these patients. Without information on stage, we are unable to confirm this. However, over time we did see that the hormone treatment rate decreased from 48% to 32%, and over this same time period the rate of surgery increased.

Woods *et al* (2006) reviewed the origins of socio-economic inequalities in cancer survival and found many studies that showed differential treatment between socio-economic groups. We also found that men from more deprived areas were less likely to receive radical treatments and more likely to receive hormone treatment. For prostate cancer there is a lower likelihood of asymptomatic screening by GPs in less advantaged areas and men from more deprived areas are less likely to be offered radical treatments.

### Other studies

Radical treatment for prostate cancer can cause serious side effects that will affect quality of life, such as urinary, bowel and sexual dysfunction; therefore, it is important to assess which treatment is optimal for men with prostate cancer. A few studies have compared survival from different treatment groups in terms of prostate cancer-specific survival and overall survival. A Scandinavian trial, which compared radical prostatectomy and watchful waiting, found that radical prostatectomy reduces disease-specific mortality and overall mortality after 10 years (Bill-Axelson *et al*, 2005). A study, which compared prostatectomy, brachytherapy and no definitive treatment, found that men treated with either the prostatectomy or brachytherapy had better survival (Tward *et al*, 2006). Other case series studies have shown that biochemical failure rates are similar for radical prostatectomy, brachytherapy and external beam radiotherapy (Kupelian *et al*, 2004; Potters *et al*, 2004). The Prostate Cancer Outcomes Study found that after 5 years men treated by radical prostatectomy experienced worse urinary incontinence than men treated with external beam radiotherapy; however, both groups had similar overall sexual dysfunction (Potosky *et al*, 2004). Other studies have assessed health-related quality of life, such as sexual functioning, urinary incontinence and bowel functioning, and found that this varies by treatment (Buron *et al*, 2007; Litwin *et al*, 2007; Ferrer *et al*, 2008; Sanda *et al*, 2008).

### Network differences in treatment received

We found that there were differences between the Cancer Networks in the treatment men received. This will be partly because of the local availability of the treatment options – brachytherapy was only available in Leeds and Newcastle during our study period. The absence of any clarity about the right treatment for some men inevitably leads to variation in practice.

Another factor that may influence treatment decisions and effect on the geographic inequalities that we observed are the waiting times for treatments such as radiotherapy and surgery. Some of the differences between networks in the treatment rates that we observed may be because of differences in waiting times. The NHS Cancer Plan published in 2000 (Department of Health, 2000) set out standards for cancer waiting times, including a 31-day standard from diagnosis/decision to treat to first treatment. The Cancer Reform Strategy (Department of Health, 2007) extended this standard to cover all cancer treatments. The largest effect that this will have is on radiotherapy delivery, in which increased capacity will be needed in some areas, and the implementation date for the application of this target to patients who receive radiotherapy will be consequently delayed. If all cancer networks are able to meet this standard, then the patient's choice of treatment should be less influenced by treatment delays.

In the United Kingdom, the Prostate Testing for Cancer and Treatment (ProtecT) trial has been set up to evaluate treatments for localised prostate cancer (http://www.epi.bris.ac.uk/protect/). This study invites asymptomatic men aged 50–69 to test for prostate cancer using PSA testing. Those who have raised age-related PSA levels are offered a transrectal ultrasound-guided biopsy and, when they are found to have localised prostate cancer, they are asked to consent to a three-armed treatment trial of prostatectomy, radiotherapy or active monitoring. Those who did not consent to randomisation could select one of the treatments. Recruitment began in 2001 and continued until 2008, with follow-up planned for 10–15 years. Studies such as this will provide vital information about which treatment is best for men with localised prostate cancer. Over the study period in our analysis, Newcastle was the major recruiting centre for this study with Leeds joining around 2005, and hence some of the men in our study would have been involved in this study. Results so far show that patients who self-selected active monitoring were more affluent than those randomised to that treatment (Mills *et al*, 2006). Levels of PSA testing are more common in more affluent areas of England and Wales (Melia *et al*, 2004; Rowan *et al*, 2008), and there are associations between affluence, PSA testing and treatment choices.

One of the key goals of the recent Department of Health Cancer Reform Strategy is to reduce inequalities in cancer incidence, access to services and outcomes (Department of Health, 2007). Inequalities in prostate cancer incidence have been reported for England with higher incidence rates observed in the most affluent areas (National Cancer Intelligence Network, 2008). Much of the recent increase in prostate cancer incidence is because of the increased use of PSA testing, resulting in the diagnosis of many asymptomatic cancers that would never previously have been diagnosed in life. There are wide variations in rates of PSA testing by GP practice (Gavin *et al*, 2004) and there is evidence that PSA testing is more common in more affluent areas in England and Wales (Melia *et al*, 2004; Rowan *et al*, 2008). There are also inequalities in prostate cancer survival and the deprivation gap in survival has increased between the late 1980s and late 1990s (Rowan *et al*, 2008), although much of this will be influenced by variations in PSA testing. We have also found socio-economic inequalities in the treatment received for men diagnosed with prostate cancer.

## Conclusions

This study highlights the wide variation that exists in the management of non-metastatic prostate cancer in the Northern and Yorkshire region of England. We have used population-based cancer registry data and found that the principal method of treatment received was found to vary by age at diagnosis, cancer network of residence and deprivation quintile.

## Figures and Tables

**Table 1:** European age-standardised rates (ASR) per 100 000 male population and 95% confidence intervals (CI) by year for all ages

**Year**	**ASR**	**95% CI**
2000	83.1	(80.2–86.0)
2001	92.9	(89.9–96.0)
2002	91.3	(88.3–94.3)
2003	95.7	(92.6–98.8)
2004	97.8	(94.7–100.9)
2005	96.7	(93.7–99.8)
2006	96.6	(93.6–99.7)
All years	93.5	(92.4–94.6)

**Table 2:** Number and percentage of men receiving each treatment

**Treatment**	***n***	**%**	**Rate per 100 000 male population**
Radical prostatectomy	2240	10.5	9.9
Brachytherapy	440	2.1	1.9
External beam radiotherapy	3410	16.0	15.1
Hormone therapy	8987	42.1	39.8
No treatment	6257	29.3	27.7

**Table 3:** Demographic characteristics of the study population by treatment group

	**Radical prostatectomy**		**Brachytherapy**		**External beam radiotherapy**		**Hormone therapy**		**No treatment**		
**Characteristic**	***n***	**%**	***n***	**%**	***n***	**%**	***n***	**%**	***n***	**%**	***P-*value** a
*Diagnosis year*											
2000	181	7.0	41	1.6	371	14.4	1231	47.9	748	29.1	<0.0001
2001	233	8.0	74	2.5	450	15.5	1356	46.7	792	27.3	
2002	280	9.4	51	1.7	400	13.4	1441	48.3	813	27.2	
2003	331	10.7	52	1.7	459	14.8	1393	45.0	859	27.8	
2004	409	12.5	87	2.7	538	16.5	1286	39.4	940	28.8	
2005	396	12.2	75	2.3	594	18.3	1233	37.9	956	29.4	
2006	410	12.6	60	1.8	598	18.3	1047	32.1	1149	35.2	
											
*Age group*											
55–64 years	1305	58.3	242	55.0	1180	34.6	740	8.2	887	14.2	<0.0001
65–74 years	870	38.8	193	43.9	1972	57.8	3097	34.5	2453	39.2	
75+ year	65	2.9	5	1.1	258	7.6	5150	57.3	2917	46.6	
											
*Network*											
YCN	1128	13.5	277	3.3	1530	18.4	2953	35.5	2440	29.3	<0.0001
HYC	277	7.8	59	1.7	783	22.1	1532	43.2	895	25.2	
NECN	835	8.8	104	1.1	1097	11.6	4502	47.6	2922	30.9	
											
*Deprivation quintile*											
1 (most affluent)	576	13.2	137	3.1	794	18.1	1525	34.9	1343	30.7	<0.0001
2	550	11.7	126	2.7	852	18.1	1832	39.0	1342	28.5	
3	468	11.4	93	2.3	635	15.4	1714	41.7	1201	29.2	
4	327	8.4	51	1.3	546	14.0	1866	47.8	1117	28.6	
5 (most deprived)	319	7.5	33	0.8	583	13.8	2050	48.4	1254	29.6	

Abbreviations: HYC=Humber and Yorkshire Coast Cancer Network; NECN=North of England Cancer Network; YCN=Yorkshire Cancer Network.

a Chi-squared *P*-value.

**Table 4:** Association between treatment received and demographic variables, odds ratios (OR) and 95% confidence intervals (CI). Results from multivariate logistic regression

	**Radical prostatectomy**		**Brachytherapy**		**External beam radiotherapy**		**Hormone therapy**		**No treatment**		**All radical treatment**	
**Characteristic**	**OR**	**95%CI**	**OR**	**95% CI**	**OR**	**95% CI**	**OR**	**95% CI**	**OR**	**95% CI**	**OR**	**95% CI**
*Diagnosis year*												
2000	1.00	—	1.00	—	1.00	—	1.00	—	1.00	—	1.00	—
2001	1.05	(0.85–1.30)	1.48	(1.00–2.18)	1.03	(0.88–1.21)	1.01	(0.90–1.13)	0.93	(0.83–1.05)	1.10	(0.96–1.27)
2002	1.35	(1.09–1.66)	1.00	(0.66–1.53)	0.86	(0.73–1.01)	1.06	(0.95–1.19)	0.93	(0.82–1.04)	1.02	(0.89–1.18)
2003	1.47	(1.20–1.79)	0.91	(0.60–1.39)	0.96	(0.82–1.12)	0.94	(0.84–1.05)	0.96	(0.86–1.08)	1.16	(1.01–1.33)
2004	1.67	(1.36–2.02)	1.32	(0.90–1.93)	1.01	(0.87–1.18)	0.77	(0.69–0.87)	1.03	(0.92–1.16	1.37	(1.19–1.57)
2005	1.55	(1.27–1.89)	1.15	(0.78–1.70)	1.15	(0.99–1.33)	0.73	(0.66–0.82)	1.07	(0.95–1.20)	1.43	(1.24–1.63)
2006	1.53	(1.26–1.86)	0.84	(0.56–1.26)	1.09	(0.94–1.26)	0.57	(0.51–0.64)	1.42	(1.27–1.59)	1.30	(1.14–149)
												
*Age group*												
55–64 years	1.00	—	1.00	—	1.00	—	1.00	—	1.00	—	1.00	—
65–74 years	0.27	(0.24–0.29)	0.40	(0.33–0.49)	0.80	(0.74–0.87)	2.72	(2.48–2.98)	1.59	(1.46–1.74)	0.32	(0.29–034)
75+ years	0.02	(0.01–0.02)	0.01	(0.004–0.03)	0.08	(0.07–0.10)	7.67	(7.00–8.41)	2.15	(1.98–2.35)	0.02	(0.02–0.03)
												
*Network*												
YCN	1.00	—	1.00	—	1.00	—	1.00	—	1.00	—	1.00	—
HYC	0.54	(0.47–0.63)	0.54	(0.40–0.71)	1.35	(1.22–1.50)	1.36	(1.25–1.49)	0.81	(0.74–0.88)	0.87	(0.79–0.97)
NECN	0.64	(0.58–0.71)	0.36	(0.28–0.45)	0.58	(0.53–0.63)	1.64	(1.53–1.75)	1.09	(1.02–1.17)	0.45	(0.41–0.48)
												
*Deprivation quintile*												
1 (most affluent)	1.00	—	1.00	—	1.00	—	1.00	—	1.00	—	1.00	—
2	0.94	(0.82–1.08)	0.92	(0.71–1.18)	1.00	(0.89–1.12)	1.17	(1.06–1.28)	0.90	(0.82–0.99)	0.95	(0.86–1.05)
3	0.96	(0.84–1.10)	0.80	(0.61–1.05)	0.84	(0.74–0.95)	1.28	(1.17–1.41)	0.92	(0.84–1.01)	0.81	(0.73–0.91)
4	0.75	(0.64–0.87)	0.55	(0.39–0.76)	0.86	(0.76–0.97)	1.50	(1.37–1.65)	0.86	(0.78–0.95)	0.70	(0.62–0.78)
5 (most deprived)	0.64	(0.55–0.75)	0.32	(0.22–0.47)	0.83	(0.74–0.94)	1.56	(1.42–1.71)	0.91	(0.83– 1.00)	0.60	(0.54–0.68)

Abbreviations: HYC=Humber and Yorkshire Coast Cancer Network; NECN=North of England Cancer Network; YCN=Yorkshire Cancer Network.
